# Horizontal Foot Speed During Submaximal and Maximal Running

**DOI:** 10.5114/jhk/159578

**Published:** 2023-04-20

**Authors:** Kenneth P. Clark, Laurence J. Ryan, Christopher R. Meng, David J. Stearne

**Affiliations:** 1 Department of Kinesiology, West Chester University of PA, USA.; 2 Independent Researcher, Dallas, TX, USA.

**Keywords:** bipedal gait, locomotor control, running biomechanics, sprinting, kinematics

## Abstract

Horizontal foot speed is fundamental for running synchronization and stability, and may also be important for sprinting performance. In this investigation, we quantified the following during steady-speed running: (a) peak forward foot speed during the swing phase, (b) backward foot speed at touchdown, and (c) ground speed difference (GSD), i.e., the difference between forward running speed and backward foot speed at touchdown. We hypothesized that forward and backward foot speed would be significantly and positively correlated with top speed, and that GSD would be significantly and negatively correlated with top speed. Participants (20 male, 20 female) completed 40-m submaximal and maximal-effort running trials, with kinematic data collected from 31–39 m. Across top speed trials, forward foot speed (r = 0.90, p < 0.001) and backward foot speed (r = 0.85, p < 0.001) were significantly and positively correlated with running speed. However, counter to expectations, GSD values slightly increased with top speed (r = 0.36, p = 0.027). These findings indicate that forward and backward foot speeds are important variables for sprinting performance, but faster runners may not necessarily exhibit lower GSD values at top speed.

## Introduction

From a kinematic perspective, faster upright running speeds are associated with greater step rates and step lengths (Dorn et al., 2012; Nummela et al., 2007; Weyand et al., 2000) and larger thigh angular amplitudes and frequencies (Clark et al., 2020). Because joint angular rotation results in segment linear translation, and since the foot is the distal segment of the leg, greater thigh angular velocities and accelerations associated with faster running speeds (Clark et al., 2021) should translate to faster foot segment motion. The term “speed” in this manuscript indicates the horizontal component of this motion. While useful terms such as “foot speed” and “turnover” are commonly utilized in coaching to describe an athlete’s ability to move the lower limbs quickly, evaluation of horizontal foot speed in the sprint biomechanics literature is relatively sparse. Further investigation of horizontal foot speed, both forward during the swing phase and in retraction prior to touchdown, may provide insight into stability and performance during submaximal and maximal upright running.

**Figure 1:**
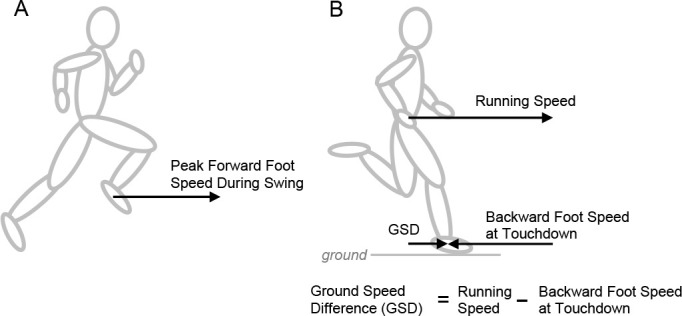
Horizontal foot speed measurements. (A) Peak forward foot speed was measured relative to the ground as the foot segment relocated in front of the body during the swing phase. (B) Running speed was quantified from the average center of mass (COM) forward speed. Backward foot speed at touchdown was measured relative to the runner, indicating how fast the foot segment was moving backward towards the COM at touchdown. Ground speed difference (GSD) was measured as the difference between the running speed and the backward foot speed at touchdown.

The synchronization requirements of steady-speed running clearly present kinematic demands for the swing foot, since it must relocate in front of the body fast enough in preparation for the next stance phase. During steady-speed running, the average forward speed over one gait cycle of any single anatomical landmark or body segment must equal the center of mass (COM) forward speed (i.e., running speed). Thus, if the foot is mostly stationary on the ground during the stance phase, its peak forward speed during the swing phase must exceed the runner’s speed for its average during the gait cycle to equal running speed (Vujošević et al., 2018). Investigating how fast the foot travels forward during the swing phase (Figure 1A) may offer insight into the coordination and synchronization requirements of swing leg movements during upright running.

After the leg protracts during the middle of the swing phase to move the foot forward in front of the body, the leg then retracts prior to touchdown (Seyfarth et al., 2003). Leg angular retraction prior to touchdown has been proposed as a feedforward control strategy to enhance stability (Seyfarth et al., 2003), and angular retraction velocity has been demonstrated as a contributing factor for top speed sprint performance (Clark et al., 2020; Mendiguchia et al., 2021; Miyashiro et al., 2019; Sinclair et al., 2013). Leg retraction results in backward movement of the foot relative to the COM prior to touchdown (Seyfarth et al., 2003) (Figure 1B). Values for backward foot speed at touchdown have been previously published during sprinting (Haugen et al., 2018; Mann and Murphy, 2018; Murphy et al., 2021), but further investigation may provide insight into how values for this variable change across a range of submaximal and maximal steady running speeds in a heterogenous sample of runners.

Another kinematic variable of interest is ground speed difference (GSD), which refers to the difference between the runner’s forward speed and the backward foot speed at touchdown (Blum et al., 2011; Fenn, 1930; Karssen et al., 2011) (Figure 1B). It has been suggested that smaller values of GSD may be beneficial for running stability and performance, resulting in reduced horizontal braking impact forces, decreased energy losses, and lower likelihood of slipping during ground contact (Blum et al., 2011; Hay, 1994; Karssen et al., 2011; Mann and Murphy, 2018). Accordingly, examining the extent to which GSD values can be minimized at faster running speeds may have practical applications for researchers and coaches interested in sprinting performance.

In this study, we examined horizontal foot speed during submaximal and maximal running trials in a heterogenous sample of participants. Specifically, the kinematic variables of interest were peak forward foot speed during the swing phase, backward foot speed at touchdown, and GSD. We hypothesized that both peak forward foot speed during the swing phase and backward foot speed at touchdown would be significantly and positively correlated with top speed, and that GSD would be significantly and negatively correlated with top speed.

## Methods

### Participants

A total of 40 athletically active participants volunteered and provided written informed consent in accordance with the local University Institutional Review Board which had approved the study. The study was conducted in accordance with the Declaration of Helsinki. Participants included 20 males (mean ± standard deviation [SD], age: 21.6 ± 2.2 years, body height: 1.80 ± 0.06 m, body mass: 79.7 ± 13.4 kg) and 20 females (age: 21.7 ± 1.8 years, body height: 1.67 ± 0.08 m, body mass: 59.0 ± 6.4 kg) from a range of athletic backgrounds. This included 15 participants from athletics (track and field), 13 participants from team sports, and 12 recreationally trained athletes.

### Design and Procedures

#### Experimental Protocol

The experimental methods have also been described in prior publications (Clark et al., 2020, 2021). Details of the experimental protocol relevant to the current study are provided below. Testing was performed in an indoor facility which included a 60–m running lane equipped with a motion capture system (eight OptiTrack Prime 13 cameras with Motive software from NaturalPoint, Corvallis, OR). Participants performed 40–m running trials with three-dimensional kinematic data obtained from 31–39 m. Each participant wore 12 reflective markers with six markers placed on the lateral aspect of each side of the body. The marker identifications and anatomical locations included: ball (running shoe area over the fifth metatarsal head), heel (running shoe area over the lateral calcaneus posterior to the peroneal tubercle), ankle (lateral malleolus), knee (lateral femoral condyle), hip (greater trochanter), and shoulder (acromion process).

Participants performed running trials at increasingly greater intensities, with the final trial at maximal intensity. For submaximal trials, participants gradually accelerated for 25 m and then ran at constant speed from 25 through 40 m. For maximal trials, participants sprinted as fast as possible for the entire 40 m. Data were captured and analyzed on four complete trials. Six of the 40 participants had submaximal trials with marker occlusions, resulting in a total of 154 trials included in the data analysis with speeds ranging from 3.1 to 10.0 m/s.

### Data Processing and Analysis

Marker kinematics were acquired at a sampling rate of 200 Hz and low-pass filtered at 25 Hz. A seven-segment model (foot, shank, and thigh on both legs, and a head-arms-trunk segment) was generated from the 12 markers and values of the COM position were determined from the marker and segment data (Winter, 2009). The foot segment was used for determining the foot horizontal position. Foot segment and COM velocities (speeds) were calculated from differentiation of the position vs. time data using the central difference method. For each trial, the horizontal foot speed variables were determined from the average of the right and left foot segment values measured during one gait cycle. Running speed was quantified from the average COM forward speed in the field of view.

The instants of the foot touchdown and takeoff were identified by reviewing all trials using the motion capture software (all analyses completed by a single investigator [first author]). The procedure involved visual inspection of the foot motion with frame-by-frame monitoring of the vertical coordinates of the ball and heel markers relative to the ground. Each analyzed trial included one full stride consisting of a left and a right step, with the complete stance and flight phases established for each step.

Peak forward foot speed was measured relative to the ground as the foot segment relocated in front of the body during the swing phase (Figure 1A). Backward foot speed at touchdown was quantified relative to the runner, indicating how fast the foot segment was moving backward towards the COM at touchdown (Figure 1B). GSD was quantified as the difference between the runner’s forward speed and the backward foot speed at touchdown (Figure 1B). In the motion capture coordinate system, GSD is equivalent to the horizontal foot speed at touchdown measured relative to the ground. Small GSD values indicate that the backward foot speed at touchdown is closer to matching the runner’s forward speed, whereas large GSD values indicate a greater difference between the runner’s forward speed and the backward foot speed at touchdown. GSD values of zero would indicate ground speed matching where the runner’s forward speed and the backward foot speed at touchdown are equivalent (Blum et al., 2011).

### Statistical Analysis

The kinematic variables of interest were peak forward foot speed during the swing phase, backward foot speed at touchdown, and GSD. To evaluate the hypotheses for the top speed trials (*n* = 40, one trial per participant), the relationship between top speed and each of these kinematic variables was evaluated using the Pearson’s *r* correlation coefficient, as well as simple linear regression with a best-fit equation (with *x* representing running speed).

Prior to the completion of the correlational or linear regression analyses, outlier data points were identified using *z*-scores. Top speed data points from one participant were identified as outliers for backward foot speed at touchdown (*z*-score = 2.95) and GSD (*z*-score = 3.55). These outlier data points were still presented in Figure 3 (see square symbols in Figures 3H and 3I), but were not included in the correlational or linear regression analyses. After the removal of outlier data points for the top speed trials, the assumptions of linear regression were checked and met.

Additionally, the ratios of peak forward foot speed to running speed and backward foot speed at touchdown to running speed were calculated for all trials. For all statistical analyses, values were expressed as mean ± standard deviation (SD).

The *a priori* threshold for all significance tests was set at *α* = 0.05. Power analysis was completed using G*Power (version 3.1.9, Kiel, Germany), based on *α* = 0.05, β = 0.8 and moderate effect size. All other statistics were completed using Microsoft Excel and GraphPad Prism software (version 9.0.1, San Diego, CA).

## Results

**Figure 2:**
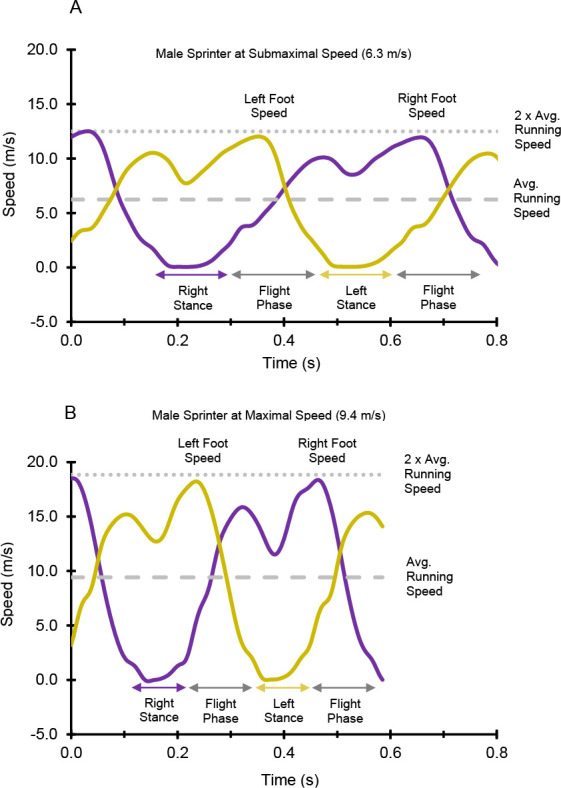
Horizontal foot speed vs. time for the left foot and right foot of a representative participant (male sprinter). Reference lines are indicated for the average running speed, twice the average running speed, stance phase time periods, and flight phase time periods. (A) Submaximal running speed of 6.3 m/s. (B) Maximal running speed of 9.4 m/s.

**Figure 3:**
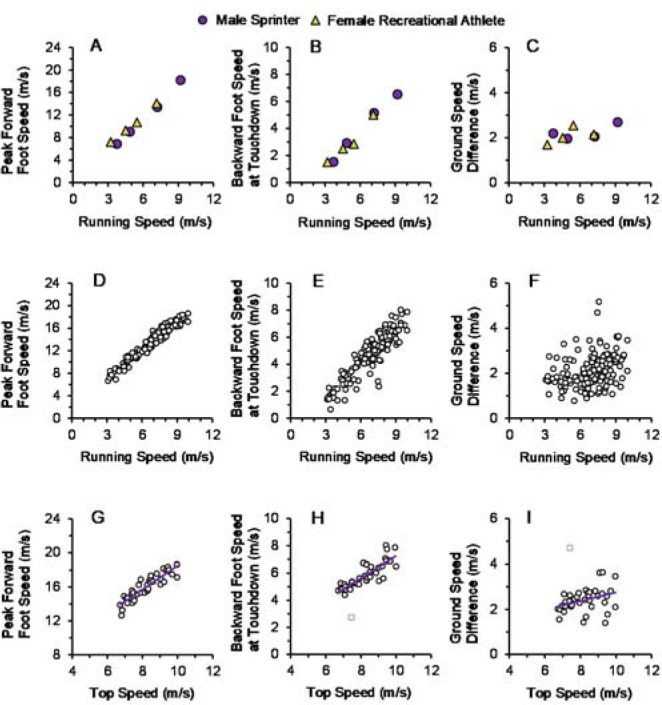
Measurements of peak forward foot speed during the swing phase, backward foot speed at touchdown, and ground speed difference. (A–C) Individual data for two representative participants across their respective range of speeds. (D–F) Data for all participants and trials (*n* = 154). (G–I) Data for the subset of top speed trials (*n* = 40, one trial per participant). Top speed data points from one participant were identified as outliers for backward foot speed at touchdown (panel H) and ground speed difference (panel I), presented as square symbols. For panels G–I, Pearson’s *r* correlation coefficients, *p*-values, and the trendline best-fit equations are summarized in the Results section.

Figure 2 displays time-series data from an individual participant (male sprinter, body height = 1.76 m, body mass = 75.0 kg). Figure 2A presents horizontal foot speed vs. time data at submaximal running speed, and Figure 2B presents horizontal foot speed vs. time data at maximal running speed. For Figure 3, the following variables are presented: peak forward foot speed during the swing phase (Figures 3A, 3D, 3G), backward foot speed at touchdown (Figures 3B, 3E, 3H), and GSD (Figures 3C, 3F, 3I). Figures 3A, 3B and 3C present data for two representative participants, a female recreational athlete (body height = 1.63 m, body mass = 61.5 kg) and another male sprinter (body height = 1.85 m, body mass = 76.7 kg), across their respective range of speeds. Figures 3D, 3E, and 3F present data for all participants and trials (*n* = 154). Figures 3G, 3H, and 3I present data for the subset of top speed trials (*n* = 40, one trial per participant).

Peak forward foot speed during the swing phase significantly increased with running speed across top speed trials (*r* = 0.90; *p* < 0.001; Figure 3G; *y* = 1.41 *x* + 4.43 [where *x* denotes running speed]). Similarly, backward foot speed at touchdown also significantly increased with running speed across top speed trials (*r* = 0.85; *p* < 0.001; Figure 3H; *y* = 0.80 *x* – 0.80). GSD values showed a small but significant increase with running speed across top speed trials (*r* = 0.36; *p* = 0.027; Figure 3I; *y* = 0.19 *x* + 0.81).

**Figure 4:**
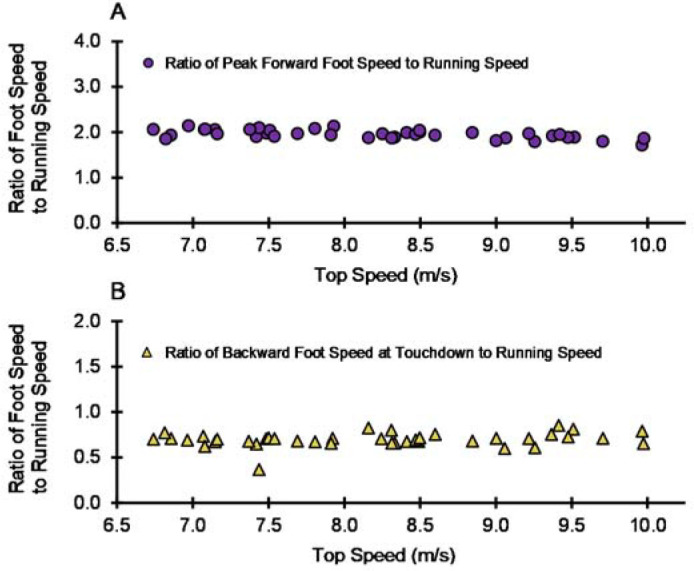
The ratio of foot speed to running speed for the top speed trials (*n* = 40, one trial per participant). (A) The ratio of peak forward foot speed to running speed for top speed trials (mean ± SD: 1.95 ± 0.10). (B) The ratio of backward foot speed at touchdown to running speed for top speed trials (mean ± SD: 0.70 ± 0.08).

The ratio of peak forward foot speed to running speed was 2.00 ± 0.15 (mean ± SD) for all participants and trials and 1.95 ± 0.10 for top speed trials. The ratio of backward foot speed at touchdown to running speed was 0.68 ± 0.12 for all participants and trials and 0.70 ± 0.08 for top speed trials. For visual purposes, the ratio data for top speed trials are presented in Figures 4A and 4B. None of the participants were able to achieve ground speed matching (GSD = 0), indicating that, for all trials, forward running speed was greater than backward foot speed at touchdown.

## Discussion

The purpose of this investigation was to evaluate horizontal foot speed during upright steady-speed running. We hypothesized that peak forward foot speed during the swing phase and backward foot speed at touchdown would be significantly and positively correlated with top speed, and that GSD would be significantly and negatively correlated with top speed. To evaluate this hypothesis, we tested a heterogeneous group of 40 participants that included males and females of different sizes, athletic backgrounds, and sprinting ability. The results for peak forward foot speed, backward foot speed at touchdown, and GSD are discussed below.

### Peak Forward Foot Speed

The first part of the hypothesis was supported by the results, as peak forward foot speed during the swing phase increased in a linear manner with running speed for individual participants (Figure 3A) as well as across all speeds (Figure 3D) and top speeds (Figure 3G). This included a strong correlation (*r* = 0.90) between peak forward foot speed and running speed across top speed trials.

Across all trials, and across the subset of top speed trials, the mean ratio of peak forward foot speed to running speed was approximately 2.0 (Figure 4A). Our findings agree with the limited prior observations on this topic. Initial data on this variable were presented by Dillmann (1974), who found that the peak forward foot speed was slightly greater than twice running speed for two participants sprinting at approximately 8 m/s. A similar statement was offered by Cross (1999), who proposed that the swing foot must reach peak forward speeds of approximately twice the COM forward speed during upright running. Further supporting evidence comes from data collected on Usain Bolt in 2011, with peak forward foot speed reported to be greater than 23 m/s in a 100 m sprint where Bolt’s top speed exceeded 12 m/s (Coh et al., 2018).

While this part of the hypothesis was supported by the data, and it is perhaps not surprising that peak forward foot speed increased with running speed, alternative values other than twice running speed were theoretically possible with different foot speed profiles or different duty factors of the stance and swing phases. Furthermore, these findings have important implications for the synchronization requirements of bipedal steady-speed running. Since the foot is the distal segment of the leg, and because segment linear translation is a result of joint angular rotation, faster running speeds should require greater joint angular velocities and joint angular accelerations. This concept has recently been demonstrated specifically for the hip joint, with thigh segment angular velocity and angular acceleration increasing linearly with running speed (Clark et al., 2020, 2021). Our data indicate that increases in running speed require proportional increases in peak forward foot speed in order to successfully reposition the foot from behind the COM to in front of it during the swing phase.

This prompts interesting questions regarding the mechanical requirements for top speed sprinting. Clearly, based on Newtonian mechanics, the instantaneous motion of a runner’s COM is determined by ground reaction force and impulse relative to body mass (along with gravity and air resistance), and these kinetic variables have been empirically established as primary determinants of sprinting performance (Clark and Weyand, 2014; Morin et al., 2012; Rabita et al., 2015; Weyand et al., 2000). However, while the motion of the COM is determined by these kinetic variables, it appears that the foot must be able to achieve a peak forward speed approaching twice the COM forward speed to maintain a stable running gait and to cyclically re-establish the stance phase in a coordinated manner at any given submaximal or maximal intensity.

### Backward Foot Speed at Touchdown and Ground Speed Difference

The second part of the hypothesis was also supported by the results, as backward foot speed at touchdown increased in a linear manner with running speed for individual participants (Figure 3B) as well as across all speeds (Figure 3E) and top speeds (Figure 3H). This included a strong correlation (*r* = 0.85) between backward foot speed at touchdown and running speed across top speed trials.

In this investigation, the mean ratio of backward foot speed at touchdown to running speed was approximately 0.7 (Figure 4B). Values from the present data set were similar to previously published group mean ratios of backward foot speed at touchdown to running speed (Haugen et al., 2018; Mann and Murphy, 2018; Murphy et al., 2021). From a performance standpoint, our findings align with prior studies confirming that backward foot speed at touchdown is an important metric for top speed sprinting (Haugen et al., 2018; Mann and Murphy, 2018; Murphy et al., 2021).

However, the third part of our hypothesis was not supported, as faster top speeds were correlated with slightly *larger* GSD values (Figure 3I). This result was surprising because several resources have described smaller GSD values as advantageous for running stability and performance, citing potential benefits of reduced horizontal braking impact forces, decreased energy losses, and lower likelihood of slipping during ground contact (Blum et al., 2011; Hay, 1994; Karssen et al., 2011; Mann and Murphy, 2018). Therefore, our expectation that faster top speeds would be significantly correlated with smaller GSD values was not upheld.

Given that larger values of backward foot speed at touchdown were associated with faster top speeds, why didn’t faster runners also have smaller GSD values compared to their slower counterparts when running at top speed? Further examination of the statistics can provide insight as to why this generally did not occur. In this data set, backward foot speed at touchdown was approximately 0.7 x running speed (Figure 4B). Formulaically, GSD is the difference between running speed and backward foot speed at touchdown, thus GSD values would slightly increase across the range of faster running speeds. Counterintuitively, this indicates that faster top speeds may not necessarily correspond with smaller GSD values (Figure 3I). In fact, aligning with our results, GSD values ranging from approximately 2.4 to 3.7 m/s have been reported for elite male sprinters running at top speeds surpassing 11.4 m/s (Bissas et al., 2022; Mann and Murphy, 2018).

From a coaching perspective, a focus on reducing GSD may still be an effective technical cue for athletes, since backward foot speed at touchdown demonstrated a positive linear relationship with running speed, and excessive GSD values in a developmental athlete may be indicative of sub-optimal sprinting mechanics. Additionally, these findings do not preclude the possibility that longitudinal coaching interventions could result in changes in GSD values when modifying an athlete’s running technique over time. However, our results suggest that researchers and practitioners evaluating the determinants of top speed performance in an acute experimental setting should not necessarily expect faster runners to demonstrate lower GSD values.

## Conclusions

In this investigation, we explored the following during steady-speed submaximal and maximal running: (a) peak forward foot speed during the swing phase measured relative to the ground, (b) backward foot speed at touchdown measured relative to the runner, and (c) ground speed difference (GSD) measured as the difference between the running speed and the backward foot speed at touchdown.

As expected, peak forward foot speed during the swing phase and backward foot speed at touchdown were significantly correlated with top speed. The ratio of peak forward foot speed to running speed was approximately 2.0. The ratio of backward foot speed at touchdown to running speed was approximately 0.7. However, counter to our expectations, GSD values slightly increased across top speeds. These findings suggest that researchers and practitioners should focus on training interventions designed to increase peak forward foot speed during the swing phase and backward foot speed at touchdown, but faster runners may not necessarily exhibit lower GSD values at top speed.

## Author Contributions

Conceptualization: K.P.C., L.J.R. and D.J.S.; methodology: K.P.C., L.J.R. and D.J.S.; software: K.P.C. and L.J.R.; validation: K.P.C., L.J.R., C.R.M. and D.J.S.; formal analysis: K.P.C., L.J.R., C.R.M. and D.J.S.; investigation: K.P.C., L.J.R., C.R.M. and D.J.S.; resources: K.P.C. and D.J.S.; data curation: K.P.C., L.J.R., C.R.M. and D.J.S.; writing—original draft preparation: K.P.C., L.J.R., C.R.M. and D.J.S.; writing—review & editing: K.P.C., L.J.R., C.R.M. and D.J.S.; visualization: K.P.C., L.J.R., C.R.M. and D.J.S.; supervision: K.P.C. and D.J.S.; project administration: K.P.C. and D.J.S.; funding acquisition: K.P.C. and D.J.S. All authors have read and agreed to the published version of the manuscript.

## ORCID iD

Kenneth P. Clark: 0000-0002-6934-1271
